# Vertical stratification and seasonality of fruit-feeding butterfly diversity in a Neotropical dry forest

**DOI:** 10.1007/s00114-026-02107-1

**Published:** 2026-05-13

**Authors:** Bianca Santana Dias Nascimento, Gabriela de Araújo Silva, Uriel de Jesus Araújo Pinto, João Rafael Macêdo, Marina do Vale Beirão, Jhonathan de Oliveira Silva

**Affiliations:** 1 Programa de Pós-Graduação em Ecologia e Evolução, Universidade Estadual de Feira de Santana, Feira de Santana, Bahia, Brazil; 2 Colegiado de Ecologia. Senhor do Bonfim, Universidade Federal do Vale do São Francisco, Bahia, Brazil; 3 Laboratório de Ecologia de Insetos, Universidade Federal de Minas Gerais, Belo Horizonte, MG Brazil

**Keywords:** Caatinga, Climatic seasonality, Insect diversity, Lepidoptera, Plant phenology

## Abstract

Vertical stratification and climatic seasonality influence the structuring of butterfly communities in tropical forests. This study assessed how vertical stratification (canopy vs. understory) and seasonal variation affect fruit-feeding butterfly (Nymphalidae) assemblages in a Brazilian tropical dry forest (TDF), and how these patterns relate to climatic variables and food resource availability. Sampling was conducted monthly over one year in the northern Espinhaço Range, within the Caatinga domain. Twenty 25 × 4 m plots were established, each with two Van Someren-Rydon traps (understory at 1 m and canopy at ≥ 6 m). Monthly measurements included temperature, humidity, light, precipitation, and zoochoric fruit availability. We recorded 2,166 individuals from 51 species, with Biblidinae and Satyrini representing over 70% of the community. *Hamadryas februa* was dominant in the understory, while *Biblis hyperia nectanabis* prevailed in the canopy. Species composition differed between vertical strata, indicating niche partitioning likely driven by microclimatic variation. Temporal patterns also influenced diversity: species richness peaked during the hottest months, and abundance peaked in the driest month, about two months after the rainy season’s peak. Subfamilies and tribes showed distinct seasonal peaks, suggesting temporal niche partitioning and reduced intergroup competition. Our findings highlight that vertical and seasonal gradients shape butterfly communities in TDFs. The high diversity and spatial-temporal dynamics observed emphasize the ecological value of the Caatinga and the need to consider vertical structure and seasonality in conservation strategies for TDFs, highly threatened and understudied ecosystems in Brazil.

**Supplementary Information:**

The online version contains supplementary material available at 10.1007/s00114-026-02107-1.

## Introduction

Tropical dry forests (TDFs) are seasonally structured ecosystems characterized by marked climatic variability, especially in precipitation, which drives a pronounced phenological cycle in plants (Pennington et al. 2018). These forests typically exhibit lower canopies compared to rainforests, but they still exhibit considerable environmental heterogeneity that supports a diverse range of organisms, particularly insects (Silva et al. 2017a, 2017b ; Rodrigues et al. 2018; Martinez-Adriano et al. 2018). The canopy experiences more pronounced climatic variability (e.g., temperature, radiation, wind), filtering species that tolerate abiotic stress (Araujo et al. 2021), while the understory accumulates organic material and decomposing fruits, serving as a primary resource base for many invertebrates (Fischer and Kirstre 2017; Brito et al. 2021).

Among TDFs, the Brazilian Caatinga is the largest, and is among the ecosystems most affected by anthropogenic disturbances, including land-use change and desertification (Antongiovanni et al. 2020). These pressures have contributed to biodiversity loss and elevated extinction risks, particularly for endemic species (Silva et al. 2017b). Seasonal rainfall in the Caatinga regulates plant phenology, driving synchronous leaf flushing and fruiting (Pezzini et al. 2014; Silva et al. 2020), which in turn structure herbivore populations (Pennington et al. 2018; Novais et al. 2019; Freire-Jr et al. 2023). Given their low dispersal capacity and dependence on local resource pulses, herbivorous insects are especially vulnerable to climatic variability (Checa et al. 2014; Wolda 1988; Novais et al. 2019).

Variation along horizontal (e.g., geographic) and vertical (e.g., stratification) gradients acts as an environmental filter, constraining species distributions and promoting niche differentiation (Bishop et al. 2016; Leal et al. 2016; Freire-Jr et al. 2022). Vertical stratification plays a critical role in community assembly by enabling niche partitioning between canopy and understory layers, thereby promoting species coexistence (Ribeiro et al. 2012; Devries et al. 2012; Santos et al. 2017). Although vertical stratification has been well studied in rainforests (Basset et al. 2012, 2015), its role in shaping insect communities in TDFs remains understudied (Neves et al. 2014).

In this context, fruit-feeding butterflies are considered valuable ecological indicators due to their high species richness, sensitivity to microclimatic conditions, and reliance on decomposing fruits and plant sap (Devries 1987; Hamer et al. 2005; Silva et al. 2025). These butterflies belong to the Nymphalidae family, subfamilies Biblidinae, Charaxinae, Satyrinae, and part of the Nymphalinae (Devries 1987; Freire-Jr et al. 2023). Despite their ecological relevance, vertical and temporal patterns in these butterflies are poorly documented in TDFs, especially in mountainous regions of northeastern Brazil—such as the Espinhaço Range—where a mosaic of habitats and strong seasonal gradients provide a unique opportunity to investigate community assembly processes (Zacca and Bravo 2012; Lima and Zacca 2014).

Given this context, we investigated how vertical stratification (canopy vs. understory) and seasonal variation influence fruit-feeding butterfly communities in a TDF over a one-year period. We also evaluated the effects of climatic variables (temperature, humidity, light, and precipitation) and resource availability (density of zoochoric fruiting plants) on butterfly richness and abundance. We tested the following hypotheses: (i) vertical stratification promotes niche partitioning and different butterfly assemblages; (ii) species richness and abundance are positively influenced by climatic variables and fruit availability; and (iii) Nymphalidae butterfly subfamilies share the temporal niche, exhibiting aligned seasonal peaks.

## Materials and methods

### Study area

The study was conducted in the Serra da Jacobina, located in north-central Bahia, Brazil. This mountain complex, approximately 250 km in length, lies in the northernmost portion of the Espinhaço Range and is part of the Caatinga domain (Lima and Zacca 2014). Annual rainfall ranges from 477.6 to 1,129.3 mm, and mean temperatures vary between 25 °C and 29 °C (Ab’Sáber 2003; Lima and Zacca 2014. The region’s vegetation is diverse and includes rocky fields, gallery forests, hillside forests, and TDFs (Lima and Zacca 2014). Despite being within the “drought polygon”—a semi-arid region characterized by high temperatures, low rainfall and humidity, and frequent droughts (Ab’Sáber 2003)—the area has notable water potential due to the presence of perennial springs and rivers.

Sampling was conducted in a TDF located in Serra da Bananeira (10º59’22” S, 40º35’92” W), between the municipalities of Antônio Gonçalves and Pindobaçu. The vegetation is characterized by shrubby Caatinga interspersed with large trees (Kerpel et al. 2014). The site is bisected by the Aipim River, which originates from two mountain tributaries (Fig. S1). The surrounding matrix consists primarily of pastureland resulting from deforestation (Silva et al. 2025). Gold mining occurred in the 1970 s, and a single fire event was recorded in 2014, after mining activities had ceased. The forest vertical structure comprises two distinct strata. The upper stratum includes native and some exotic fruit trees, ranging from 8 to 12 m tall, with relatively dense canopies throughout most of the year. The lower stratum consists of a sparse understory composed of shrubs and small trees between 1 and 3 m tall. Light and wind penetration are reduced in the understory, and lianas and epiphytes are generally rare in both layers.

### Sampling

Fruit-feeding butterflies were sampled monthly over a 12-month period (October 2022 to September 2023). Twenty fixed plots measuring 25 × 4 m (0.01 ha each) were established, totaling 0.2 hectares. Plots were spaced at least 100 m apart, with an average distance of 250 m and a maximum of 1,560 m between them, ensuring spatial independence among sampling units (Pessoa et al. 2017). In the center of each plot, two Van Someren-Rydon (VSR) traps were installed: one in the canopy (≥ 6 m) and one in the understory (1 m) (Fig. S2), totaling 40 traps per sampling campaign (20 traps in the understory and 20 in the canopy). Following Devries et al. (2012), traps were baited with a fermented mixture of banana and sugarcane juice (6:1 L ratio, fermented for 48 h).

The 40 traps were installed monthly, in the same 20 plots (during the second or third week of each month), and remained in the field for 48 h, totaling 576 h of sampling per trap (over 36 days) in total. Captured Nymphalidae individuals were stored in labeled envelopes with all relevant metadata. Specimens were identified using Palo-Jr (2017) and Orlandin et al. (2020), with confirmations by a lepidopterist. For taxonomic consistency with previous studies, we followed a classification system grouping the subfamilies Biblidinae, Charaxinae, Nymphalinae, and Satyrinae (separating them into the tribes Brassolini, Morphini, and Satyrini), based on the greater ecological sensitivity to disturbances of the tribes Brassolini and Morphini (Devries 1987; Santos et al. 2017; Freire-Jr et al. 2023).

All woody (CBH ≥ 5 cm) and herbaceous/shrubby (CBH ≤ 4 cm) plants within the plots were tagged, totaling 1,094 individuals. Monthly data were collected on climatic variables (temperature, light, humidity, and rainfall) and resource availability, quantified as the density of zoochoric fruiting plants per plot and fruiting intensity (Pessoa et al. 2017). Plant phenology was assessed using Fournier’s Percentage Intensity method (Fournier 1974), a semi-quantitative scale from 0 to 4 in 25% intervals (Table S1). Only ripe fruits were considered, to reflect the actual availability of food resources, and peak fruiting was defined as months with the highest proportion of fruiting individuals (Pessoa et al. 2017).

Temperature, light intensity, and relative humidity were recorded in each plot during sampling using a thermohygrometer (see Freire-Jr et al. 2015). Monthly precipitation data were obtained from the A428 Meteorological Station of the Brazilian National Institute of Meteorology (INMET), located in Senhor do Bonfim, 28 km from the study site.

### Data analysis

Initially, to test the first hypothesis, we compared butterfly communities between the canopy and understory strata. Species abundance rankings were generated to identify dominant species in each vertical layer. To avoid temporal pseudoreplication, species richness and abundance were summed over the 12-month period for each trap (2 traps per plot × 20 plots; *n* = 40). A non-metric multidimensional scaling (NMDS) analysis was then performed to assess differences in community composition between strata based on species abundance, using Bray–Curtis dissimilarity and 1,000 permutations. The dissimilarity matrix was used in a non-parametric permutation procedure (PERMANOVA) with 1000 randomizations (Anderson 2001). This analysis was conducted using the *vegan* package in R (Oksanen et al. 2013).

Next, generalized linear mixed-effects models (GLMMs) were applied to test for differences in species richness and abundance between strata. The richness and abundance values ​​for each month were used as response variables, with the vertical stratum (understory or canopy) as a fixed effect (*n* = 24). The month’s ID, nested within the stratum, was included as a random effect. Models were fitted using the “glmer” function from the *lme4* package (Bates et al. 2015) assuming a Poisson distribution, more suitable for count data. Residual diagnostics were performed using the *DHARMa* package to assess model fit and distributional assumptions (Hartig et al. 2016).

To test the second hypothesis, namely the influence of climatic and biotic variables on temporal variation in butterfly diversity (abundance and richness), additional GLMMs were applied, with vertical variation (understory and canopy) included as a random effect. For these models, total species richness and abundance were summed monthly for each plot (20 plots × 12 months; *n* = 240). Explanatory variables included time (month), climatic variables (light intensity, humidity, and temperature), and resource availability (density of zoochoric fruiting plants). Plot ID nested within time (months) was included as a random effect. Model selection was based on corrected Akaike Information Criterion (AICc), and Akaike weights (wAICc) were calculated to quantify model support (Burnham et al. 2011). Candidate models with ΔAICc ≤ 2.0 and wAICc ≥ 0.90 were retained using the MuMIn package (Barton 2019). Additional GLMMs were performed to assess the effects of precipitation on butterfly diversity. For these models, species richness and abundance were summed across all plots per month (*n* = 12). Models were fitted using a Poisson distribution and validated via residual diagnostics (Hartig et al. 2016).

Circular statistics were employed to test the third hypothesis, evaluating seasonal patterns in butterfly activity (both overall and by stratum). This method is commonly used for phenological and temporal occurrence data by converting linear time into angular data (radians) to detect periodic trends (Morellato et al. 2010). Each month was represented as a 30° interval on a 360° circular scale. Observations were converted into vectors based on sine and cosine transformations to calculate the mean angle (µ), indicating the average month of occurrence, and the vector length (r), indicating the strength of seasonality. Analyses were also conducted for each subfamily or tribe (Biblidinae, Charaxinae, Nymphalinae, and Satyrinae: Brassolini, Morphini, and Satyrini). In this way, our seasonal inferences are limited to the 12-month sampling period. All statistical analyses were conducted in R version 3.6.3 (R Development Core Team 2020).

## Results

**Fig. 1:**
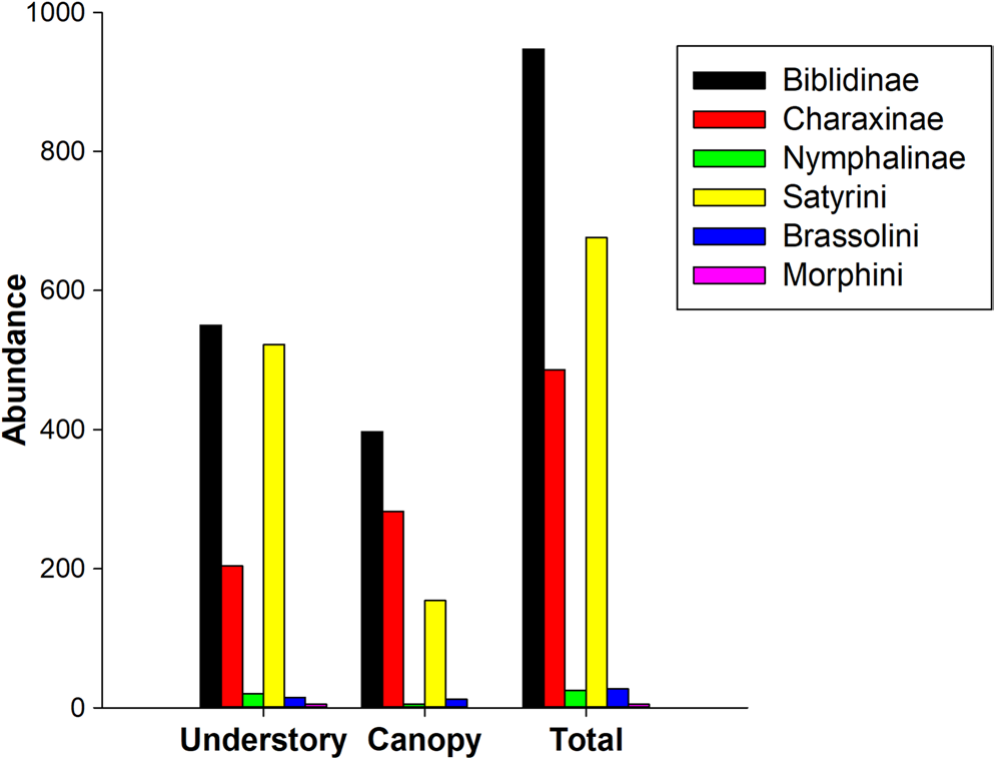
Abundance of subfamilies and tribes in the overall community and between vertical strata (understory and canopy)

We sampled 2,166 individuals belonging to 51 fruit-feeding butterfly species of the Nymphalidae family (Table 1). These species were distributed across four subfamilies: Satyrinae (25 species), Biblidinae (15 species), Charaxinae (8 species), and Nymphalinae (3 species). Within Satyrinae, four species were belonged to the Brassolini tribe, one to Morphini, and 20 to Satyrini (Table 1). Of the total butterflies collected, 1,316 individuals (60.7%) were captured in the understory and 850 (39.3%) in the canopy (Table 1). The proportional distribution of subfamilies and tribes was similar between the two strata (Fig. S3), with abundance peaks largely coinciding among them, except to Satyrini, which peaked in the understory (Fig. 1).

**Table 1:** Checklist of nymphalidae species recorded in Serra da Bananeira, Bahia, Brazil, organized by subfamily/tribe, with the respective number of individuals collected in each stratum (understory and canopy)

Subfamily/tribe/species	Abundance	
Biblidinae	Understory	Canopy
*Biblis hyperia nectanabis* (Fruhstorfer, 1909)*	64	122
*Callicore sorana* (Godart, 1824)	0	15
*Catagramma pygas* (Godart, 1824)	0	3
*Eunica tatila bellaria* (Fruhstorfer, 1908)	13	45
*Hamadryas amphinome* (Linnaeus, 1767)	1	2
*Hamadryas* sp*	10	17
*Hamadryas arete* (Doubleday, 1847)	13	16
*Hamadryas arinome arinome* (Lucas, 1853)	20	24
Hamadr*yas epinome* (Felder, 1867)	24	6
*Hamadryas februa* (Hübner, 1823)	293	75
*Hamadryas feronia* (Linnaeus, 1758)	28	25
*Hamadryas iphthime* (H. Bates, 1864)	24	6
*Myscelia orsis* (Drury, 1782)	93	6
*Paulogramma pygas* (Dillon, 1948)	2	11
*Pyrrhogyra neaerea* (Linnaeus, 1758)*	0	1
*Temenis laothoe* (Ebert, 1965)	1	2
Charaxinae		
*Archaeoprepona demophon demophon* (Linnaeus, 1758)*	1	1
*Archaeoprepona demophon thalipus* (Hübner,1814)*	3	0
*Fountainea glycerium cratais* (Doubleday, 1849)	33	46
*Fountainea halice moretta* (H. Druce, 1877)	6	7
*Fountainea ryphea* (Cramer, 1775)	2	2
*Hypna clytemnestra forbesi* (Godman & Salvin, 1884)	11	11
*Memphis_moruus* (Prittwitz, 1865)	1	0
*Zaretis strigosus* (Hübner,1819)	1	4
Nymphalinae		
*Colobura dirce dirce* (Linnaeus, 1758)	17	3
*Historis odius* (Fabricius, 1775)	0	2
*Siproeta stelenes* (Linnaeus, 1758)*	0	3
Satyrinae		
*Brassolini*		
*Caligo illioneus* (Cramer, 1775)	6	0
*Eryphanis reevesii* (Doubleday, 1849)	1	0
*Opsiphanes cassiae* (Linnaeus, 1758)	0	1
*Opsiphanes invirae* (Hübner, 1808)	8	11
*Morphini*		
*Morpho helenor* (Cramer, 1776)	5	0
*Satyrini*		
*Dynamine postverta* (Cramer, 1779)*	1	0
*Dynamine tithia* (Hübner, 1823)*	0	1
*Godartiana byses* (Godart,1824)	2	0
*Hermeuptychia sp.*	2	2
*Magneuptychia lethra* (Möschler, 1883)	14	16
*Pareuptychia ocirrhoe* (Fabricius, 1776)	7	2
*Paryphthimoides phronius* (Godart, 1824)	4	0
*Paryphthimoides poltys* (Prittwitz, 1865)	124	44
*Pharneuptychia ca. pharnobazos* (Bryk, 1953)	28	6
*Satyrinae *sp1*.*	3	2
*Satyrinae *sp2*.*	1	3
*Satyrinae *sp3*.*	5	3
*Taygetis laches* (Fabricius, 1793)	2	0
*Taygetis sylvia* (Bates, 1866)	66	28
*Taygetis thamyra* (Cramer, 1779)	80	12
*Yphthimoides affinis* (Butler, 1867)*	0	1
*Yphthimoides celmis* (Godart, 1824)	21	7
*Yphthimoides ochracea* (Butler, 1867)	69	6
*Yphthimoides pacta* (Weymer, 1911)	13	10
*Yphthimoides renata* (Stoll, 1780)	47	8

*The individuals here labeled as *Hamadryas* sp. correspond to those too damaged for species-level identification. They were not included in the richness and diversity analyses, only in the abundance analyses

*The species highlighted here with a pink asterisk have a generalist diet, feeding on nectar, fruits, and other decaying organic matter

**Fig. 2:**
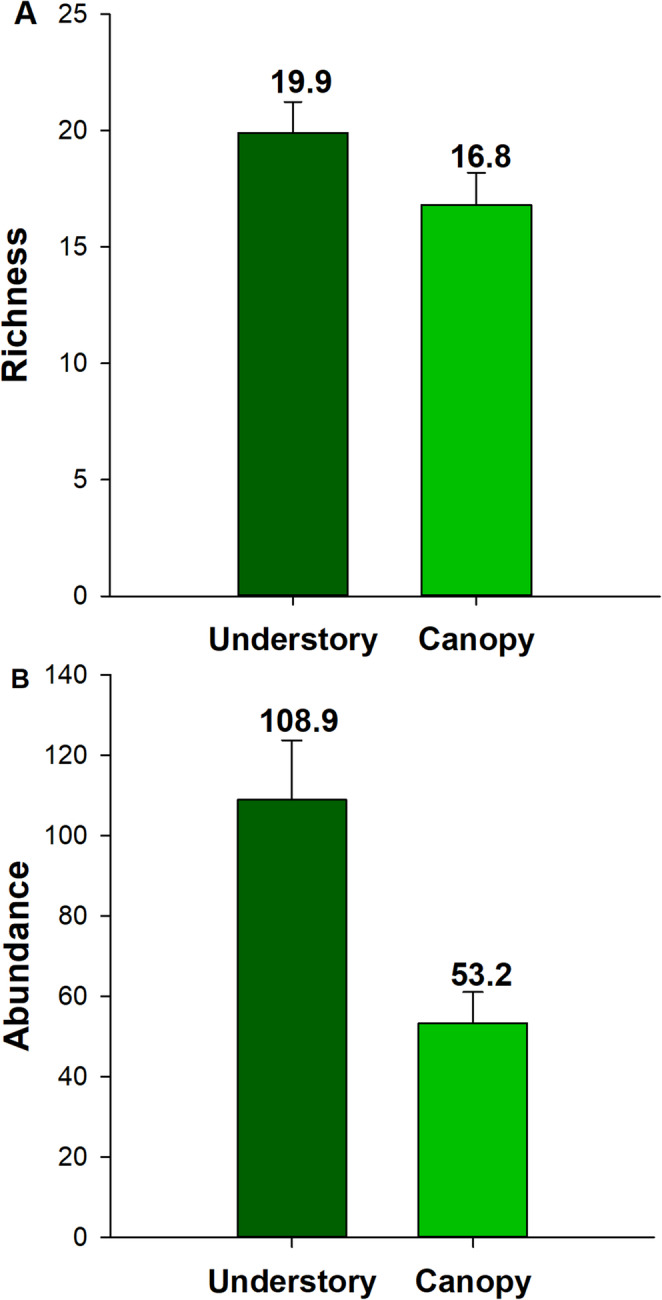
Comparison of richness (**A**) and abundance (**B**) of fruit-feeding butterflies between the vertical strata in a dry tropical forest. Data are presented as mean ± standard error (*n* = 24)

Community composition differed significantly between vertical strata (PERMANOVA: *F* = 10.37, *p* = 0.001). In total, nine species were exclusive to the understory, eight to the canopy, and 34 species occurred in both strata (Table 1). The *Hamadryas februa* (Biblidinae) was the most abundant species overall (371 individuals), with most individuals found in the understory (293; Fig. S4). The species *Biblis hyperia nectanabis* (Biblidinae) was the most abundant species in the canopy (122 individuals). While species richness did not differ significantly between strata (*X* ^2^ = 30.26, *p* = 0.07; Fig. 2A), average abundance was twofold higher in the understory (108.9 ± 14.8 individuals) compared to the canopy (53.2 ± 7.82; *X* ^2^ = 17.87, *p* = 0.001; Fig. 2B).

**Fig. 3:**
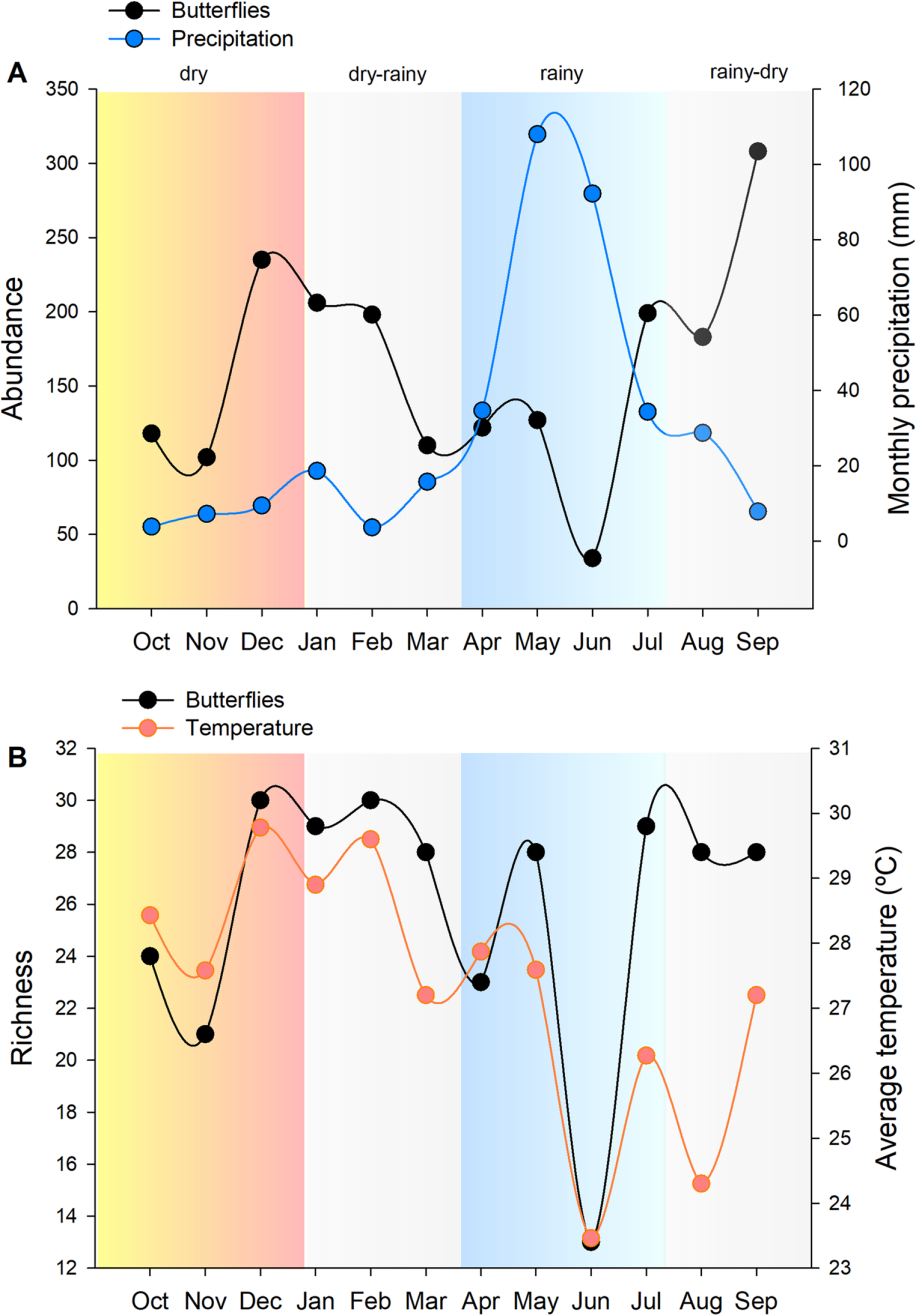
(**A**) Monthly abundance of fruit-feeding butterflies and total monthly precipitation. (**B**) Monthly species richness of butterflies and average monthly temperature. Blue shading indicates the rainy season

**Fig. 4:**
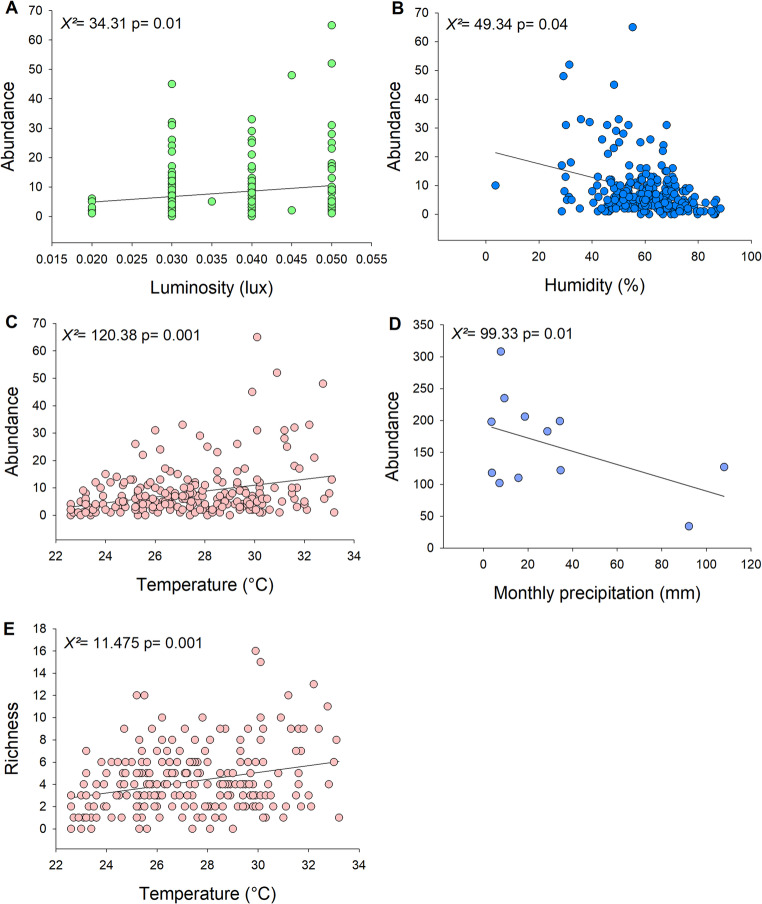
Effect of climatic variables: light intensity, humidity, temperature (*n* = 240), and precipitation (*n* = 12), on butterfly abundance, and of temperature (*n* = 240) on butterfly species richness

The local butterfly community exhibited seasonal patterns, with significant variation in abundance and richness between months (Fig. 3; Table 2). Butterfly abundance was positively influenced by temperature and light, and negatively by humidity and precipitation (Fig. 4; Table 2). The peak of total abundance (308 individuals) occurred in September, the driest month (Fig. 3A). The peak of species richness (30 species) occurred between December and February, the warmest period (Fig. 3B). Species richness was positively associated only with temperature (Fig. 4D; Table 2). Food resource availability (density of fruiting plants) did not significantly affect butterfly abundance (*χ²* = 0.12, *p* = 0.8) or richness (*χ²* = 0.002, *p* = 0.9). Overall, fruiting was sparse, with only 80 individuals (7.3%) bearing ripe fruits throughout the year, with little temporal variation.

**Table 2:** Effect of temporal variation, and climatic variables on the abundance and richness of fruit-feeding butterflies in a tropical dry forest. Legend effects: (≠) indicates significant difference; (-) indicates negative effect; and (+) indicates positive effect

Response variable	Explanatory variable	X^2^	DF	*P*	Effect
Abundance	Months	389.11	11	0.001*	≠
	Precipitation	99.33	1	0.01*	-
	Humidity	49.34	1	0.04*	-
	Luminosity	34.31	1	0.01*	+
	Temperature	120.38	1	0.001*	+
Richness	Months	88.693	11	0.001*	≠
	Temperature	11.475	1	0.001*	+

**Fig. 5:**
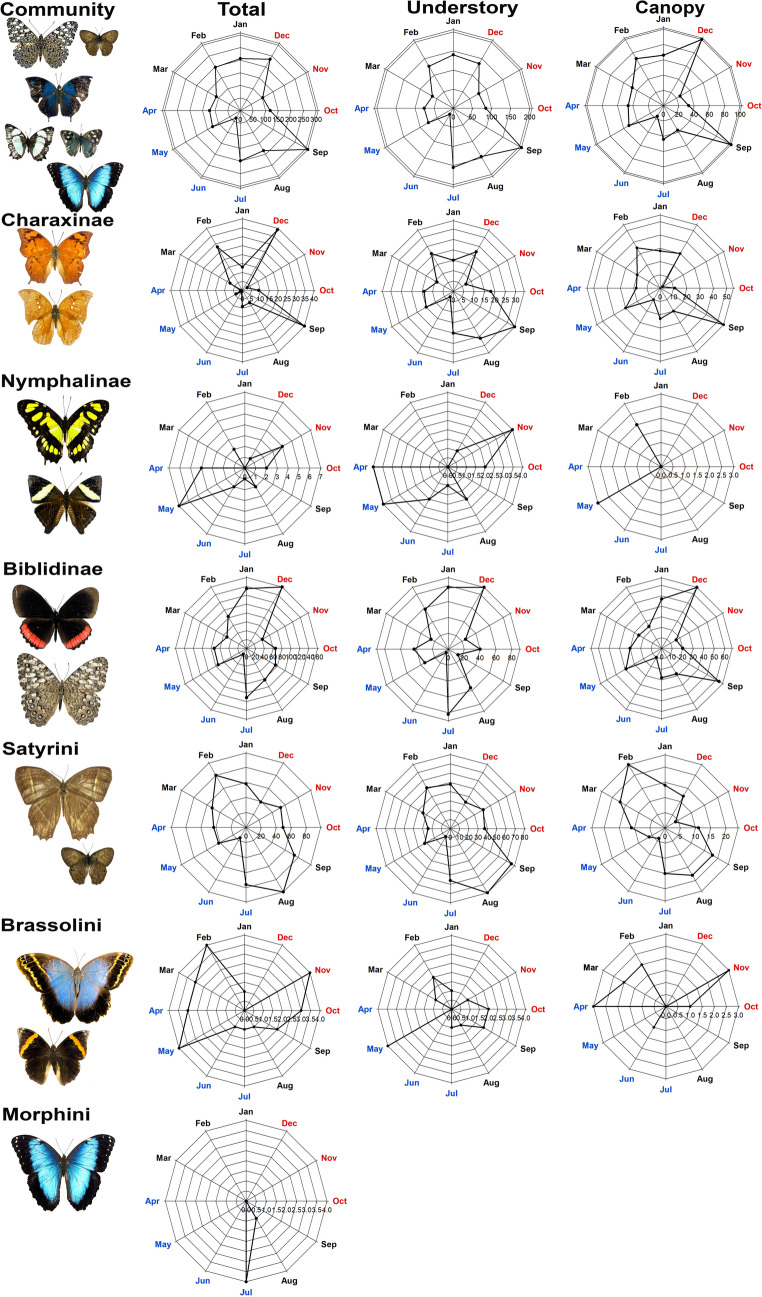
Circular distribution of fruit-feeding butterfly abundance throughout the year (October/2022 to September/2023), showing vector corresponding to total and habitat-specific abundance for the community and each subfamily/tribe. Colors represent seasonality: red = dry months; blue = rainy months; black = transitional months (dry–rainy and rainy–dry)

The circular analysis revealed that most subfamilies and tribes had a non-random temporal distribution, with a majority showing bimodal patterns (Fig. 5). The Community-level temporal patterns differed between the understory and canopy strata: the understory exhibited a unimodal distribution, peaking between July and September (38.91% of individuals), whereas the canopy showed a bimodal pattern, with peaks in December and September (15.64% and 15.96% of individuals, respectively; Fig. 5). Subfamilies and tribes also exhibited distinct seasonal abundance patterns in both strata (Fig. S3).

For the entire community and for the understory, the Biblidinae subfamily showed abundance peaks in December and July. In the canopy, peaks occurred in December and August (Fig. 5). Other subfamilies had synchronized abundance peaks between strata: Charaxinae peaked in September and December; Nymphalinae in November and May. Satyrini peaked in February and August, while in the canopy, peaks occurred in February and again from August to September. Brassolini showed a bimodal distribution with distinct peak periods across strata: (1) in the canopy, peaks occurred in November and from February to May—just before the rainy season; (2) in the understory, a single peak occurred in May, the wettest month recorded (Fig. 5). Morphini was the only tribe with a unimodal pattern, peaking in July, the month with the second highest recorded rainfall (Fig. 5). However, results for Morphini should be interpreted with caution, as the model included only a single species (five individuals), all found in the understory.

## Discussion

There were temporal changes in the richness, abundance, and distribution of fruit-feeding butterflies (Nymphalidae). As expected, the species composition differed between the understory and the canopy, probably due to vertical microclimatic variation, the canopy of the TDFs experiences more intense climatic variability (temperature, radiation, wind), filtering out species that tolerate abiotic stress (Devries et al. 2012; Araujo et al. 2021). The highest butterfly diversity was recorded during hot and dry periods, and there appears to be a time lag of approximately two months between the onset of the rainy season and a subsequent increase in butterfly abundance. We recorded 51 species, a richness similar to that reported by Lima and Zacca (2014) in the same region. This number is higher than those found in other TDFs within the Caatinga domain: Nobre et al. (2012) and Santos et al. (2023) recorded 15 and 42 species in Pernambuco, and 12 species in Rio Grande do Norte and Paraíba, respectively. These results highlight the conservation value of the Serra da Jacobina region. Additionally, the presence of species: (1) endemic to the Caatinga, such as *Fountainea halice moretta* and *Hypna clitemnestra forbesi* (Nobre et al. 2012; Zacca and Bravo 2012); and (2) rare in TDFs, such as *Archaeoprepona demophon demophon* and *A. demophon thalipus* (Santos et al. 2023), reinforces this importance.

Although both strata exhibited the same overall species richness (43 species), the composition of the butterfly community differed. Nine species were exclusive to the understory and eight to the canopy, indicating possible vertical niche partitioning. Among the species occurring in both strata, the seven most abundant in the understory had threefold lower abundances in the canopy (Table 1; Fig. S4). Only three of these species remained among the most abundant in the canopy—*Biblis hyperia nectanabis*, *Fountainea glycerium cratais*, and *Hamadryas feronia*—species known to be more tolerant of anthropogenic disturbance and microclimatic fluctuations (Orlandin et al. 2020). The understory had twice the butterfly abundance observed in the canopy, likely due to its more stable microclimatic conditions and the accumulation of organic material and decomposing fruits in the understory, serving as a food resource base (Araujo et al. 2021; Brito et al. 2021; Freire-Jr et al. 2022). The understory also provides shelter for climate-sensitive individuals, as forest cover buffers extreme conditions such as wind and rainfall, which can damage wings and hinder flight in small species (Checa et al. 2014). These findings support the hypothesis that butterfly communities are structured by vertical niche partitioning.

Climatic variables—such as humidity, precipitation, and temperature—are generally positively associated with butterfly diversity in tropical ecosystems like the Atlantic Forest, Cerrado, and TDFs (Nobre et al. 2012; Beirão et al. 2017; Martinez-Adriano et al. 2018; Lourenço et al. 2019; Santos et al. 2023). However, our findings partly diverged from these expectations: precipitation and humidity negatively affected butterfly abundance, while only temperature had a positive effect on richness (Fig. 4). The highest species richness occurred between December and February, the warmest period. Temperature is known to accelerate insect metabolism, development, and reproductive rates (Wolda 1988), which may explain its positive effect. In contrast, butterfly abundance peaked in September, the driest month, approximately two months after the rainfall peaks of May and June (Fig. 3B). This pattern likely reflects increased host plant availability for caterpillars following early rains (Pezzini et al. 2014; Silva et al. 2020), which stimulates larval development and subsequently leads to an increase in adult butterfly emergence during the dry season (Morais et al. 1999; Silva et al. 2012; Novais et al. 2019; Freire-Jr et al. 2023).

Plant fruiting density also had no significant effect on butterfly diversity, possibly due to the generalist diets of most recorded species (Orlandin et al. 2020; Santos et al. 2023). In TDFs, zoochoric plants with fleshy fruits represent only 4–13% of plant species (Pezzini et al. 2014), a sharp contrast to humid tropical forests (Freitas et al. 2013; Oliveira et al. 2018). These species fruit only briefly during the rainy season and are strongly tied to precipitation (Pezzini et al. 2014). Thus, fruit-feeding butterfly abundance did not coincide with fruiting peaks in our study area. Only three large species with diets considered more specialized were recorded: *Caligo illioneus*, *Eryphanis reevesii*, and *Morpho helenor*. Although commonly referred to as frugivorous, many Nymphalidae also feed on nectar, tree sap, plant exudates, minerals, feces, and decaying animals depending on the availability of food resources (Devries 1987; Orlandin et al. 2020; Santos et al. 2023). Most of the species recorded here are feeding generalists and likely resort to alternative food sources when their main food sources are scarce (Spaniol et al. 2019). Species considered predominantly nectarivorous, such as: *Archaeoprepona demophon demophon*, *A. demophon thalipus*, *Biblis hyperia nectanabis*, *Dynamine postverta*, *Dynamine tithia*, *Pyrrhogyra neaerea*, *Siproeta stelenes*, and *Yphthimoides affinis*, may be generalists in TDFs. The same observations were made in this and other studies conducted on TDFs feeding on fruits, feces, and decomposing animals during dry periods, demonstrating their dietary plasticity in seasonal ecosystems (see Santos et al. 2023; Silva et al., 2025).

The distribution of some subfamilies followed patterns consistent with studies in the Caatinga, Cerrado, and Atlantic Forest, in which Biblidinae and Satyrinae tribes alternate as the most species-rich and abundant groups (Beirão et al. 2017; Lourenço et al. 2019; Silva et al. 2025). The Biblidinae subfamily and the Satyrini and Brassolini tribes were the only groups to show asynchronous abundance peaks between canopy and understory, accounting for more than 70% of the entire community. These groups may shift from the understory to the canopy after peak rainfall (May and June) as a strategy to maintain dominance. Most subfamilies and tribes exhibited bimodal temporal distributions, as reported in earlier studies (Beirão et al. 2017; Freire-Jr. et al. 2023). The lack of overlap in peak abundance among the main subfamilies indicated temporal niche partitioning. The most abundant groups (Biblidinae and Satyrini), as well as Charaxinae and Nymphalinae, and Brassolini and Morphini, showed staggered peaks throughout the year (Fig. 5), suggesting that these groups may avoid interspecific competition by occupying different temporal niches for reproduction and activity.

The Satyrinae subfamily comprises species with diverse traits—such as body size, coloration, diet, hardiness, and life span—which may explain the broad and stratified distribution patterns observed among its tribes (Freire-Jr. et al. 2023). For example, the Brassolini and Morphini tribes are more sensitive to disturbance (Santos et al. 2017), so much so that the Morphini tribe occurred only during the rainy season (Fig. 5), a period with a milder climate in the TDFs. In contrast, species from other subfamilies tend to share similar traits, contributing to their more synchronized seasonal peaks. Butterfly abundance was lower in June and December, corresponding to periods of increased rainfall, humidity, and reduced temperature. Biblidinae species, however, did not follow this trend (Fig. 5). The most abundant species in this subfamily, *Biblis hyperia nectanabis* and several *Hamadryas* species, are habitat generalists (Orlandin et al. 2020; Santos et al. 2023). Traits such as dietary plasticity, as upside-down perching behavior, cryptic coloration for camouflage on tree trunks, and medium-to-large body size facilitating flight (Devries 1987; Orlandin et al. 2020) may contribute to the long-term persistence and wide temporal activity of Biblidinae species.

## Conclusion

To our knowledge, this is the first medium-to-long-term study comparing vertical stratification of fruit-feeding butterflies in a Brazilian TDF. We recorded greater diversity than in other studies in TDFs that did not sample the canopy. Vertical niche partitioning was evident, with species composition differing between canopy and understory and greater abundance observed in the understory. Richness peaked from December to January, associated with higher temperatures, while abundance peaked in September, the driest month. Temporal niche partitioning was indicated by staggered peak occurrences among subfamilies, reflecting differing life histories and ecological strategies. The high diversity and spatial-temporal differentiation of the butterfly community underscore the need to include vertical stratification in biodiversity assessments and conservation strategies. Our results also contribute to knowledge of local bioindicator insect fauna, supporting conservation efforts in the Serra da Jacobina complex, a priority region with limited protected areas. Additionally, this study can inform the development of a species guide that supports identification by researchers, taxonomists, and enthusiasts, while promoting science outreach for the general public.

## Supplementary Information

Below is the link to the electronic supplementary material.

Supplementary Material 1 (DOCX 1.54 MB)

## Data Availability

Available on request.

## References

[CR1] Ab’Sáber NA (2003) Os domínios de natureza no Brasil: potencialidades paisagísticas. Ateliê Editorial, São Paulo

[CR69] Anderson MJ (2001) A new method for non-parametric multivariate analysis of variance. Austral Ecol 26:32–46. 10.1111/j1442-9993.2001.01070.pp.x

[CR64] Antongiovanni M, Venticinque EM, Matsumoto M, Fonseca CR (2020) Chronic anthropogenic disturbance on Caatinga dry forest fragments. J Appl Ecol 57(10): 2064–2074. 10.1111/1365-2664.13686

[CR2] Araujo PF, Freitas AVL, Gonçalves GADS, Ribeiro DB (2021) Vertical stratification on a small scale: the distribution of fruit-feeding butterflies in a semi-deciduous Atlantic Forest in Brazil. Stud Neotrop Fauna Environ 56:10–39. 10.1080/01650521.2020.1728033

[CR5] Barton K (2019) Package MuMIn. R package version 1.43.6. https://cran.r-project.org/web/packages/MuMIn/index.html

[CR7] Basset Y, Barrios H, Segar S, Srygley RB, Aiello A, Warrens AD et al (2015) The butterflies of Barro Colorado Island, Panama: local extinction since the 1930s. PLoS One 10(8):1–22 10.1371/journal.pone.0136623 PMC4549329 26305111

[CR6] Basset Y, Cizek L, Cuénoud P, Didham RK, Guilhaumon F, Missa O et al (2012) Arthropod diversity in a tropical forest. Science 338:1481–1484. 10.1126/1226727 23239740 10.1126/science.1226727

[CR8] Bates D, Mächler M, Bolker BM, Walker SC (2015) Fitting linear mixed-effects models using lme4. J Stat Softw 67:1–48. 10.18637/jss.v067.i01

[CR9] Beirão MV, Neves FS, Penz CM, Devries PJ, Fernandes GW (2017) High butterfly beta diversity between Brazilian cerrado and cerrado–caatinga transition zones. J Insect Conserv 21:1–12. 10.1007/s10841-017-0024-x

[CR11] Bishop TR, Robertson MP, Gibb H, Van Rensburg BJ, Braschler B, Chown SL, Foord SF, Munyai TC, Okey I, Tshivhandekano PG, Werenkraut P, Parr CL (2016) Ant assemblages have darker and larger members in cold environments. Global Ecology and Biogeography. 10.1111/geb.12516

[CR12] Brito MRM, Lion MB, Oliveira IF, Cardoso MZ (2021) Butterflies on the dry edge of the Atlantic Forest: water availability determines community structure at the Northern limit of Atlantic Forest. Insect Conserv Divers 14:476–491. 10.1111/icad.12474

[CR13] Burnham KP, Anderson DR, Huyvaert KP (2011) AIC model selection and multimodel inference in behavioral ecology: some background, observations, and comparisons. Behav Ecol Sociobiol 65:23–35. 10.1007/s00265-010-1029-6

[CR14] Checa MF, Rodriguez J, Willmott KR, Liger B (2014) Microclimate variability significantly affects the composition, abundance and phenology of butterfly communities in highly threatened Neotropical dry forest. Fla Entomol 97:1–13. 10.1653/024.097.0101

[CR16] deAraújo Silva G, Nascimento BSD, deJesus Araújo Pinto U, dosSantos ALC, doVale Beirão M, deOliveira Silva J (2025) Changes in vegetation cover and their effects on the diversity of fruit-feeding butterflies. Austral Ecol 50:e70095. 10.1111/aec.70095

[CR17] Devries PJ (1987) Papilionidae, Pieridae and Nymphalidae. In: Devries PJ (ed) The butterflies of Costa Rica and their natural history. Princeton University Press, New Jersey, p 327

[CR18] Devries PJ, Alexander LG, Chacon IA, Fordyce JA (2012) Similarity and difference among rainforest fruit-feeding butterfly communities in Central and South America. J Anim Ecol 81:472–482. 10.1111/j.1365-2656.2011.01922.x 22092379 10.1111/j.1365-2656.2011.01922.x

[CR20] Fernandes GW (2016) Ecology and conservation of mountaintop grasslands in Brazil. Springer International Publishing, Switzerland

[CR21] Fischer K, Kirste M (2017) Temperature and humidity acclimation increase desiccation resistance in the butterfly *Bicyclus anynana*. Entomol Exp Appl 166:289–297. 10.1111/eea.12662

[CR22] Fournier LA (1974) Un método cuantitativo para la medición de características fenológicas en árboles. Turrialba 24:4

[CR24] Freire GB Jr, Salcido D, Oliveira HFM, Ribeiro DB, Provete DB, Silva T, Dias JP, Rodrigues HP, Santos JP, Diniz IR (2023) Body size and its correlates in fruit‑feeding butterflies in a seasonal environment. J Insect Conserv. 10.1007/s10841-023-00481-z

[CR23] Freire GN Jr, Nascimento AR, Malinov IK, Diniz IR (2015) Temporal occurrence of two *Morpho* butterflies (Lepidoptera: Nymphalidae): influence of weather and food resources. Environ Entomol 43:274–282. 10.1603/EN12352 10.1603/EN12352 24495483

[CR68] Freire-Jr GB, Diniz IR (2015) Temporal dynamics of fruit-feeding butterflies (Lepidoptera: Nymphalidae) in two habitats in a seasonal Brazilian environment. Fla Entomol 98(4):1207–1216. https://www.jstor.org/stable/24587636

[CR67] Freire-Jr GB, Ribeiro DB, Santos AC, Silva T, Dias JP, Rodrigues HP, Diniz IR (2022). Horizontal and vertical variation inthe structure of fruit‐feeding butterfly (Nymphalidae) assemblages in the Brazilian Cerrado. Insect Conserv Divers 15(2): 226–235. 10.1111/icad.12547

[CR25] Freitas TG, Souza CS, Aoki C, Arakaki LMM, Stefanello TH, Sartori ÂLB, Sigrist MR (2013) Flora of Brazilian humid Chaco: composition and reproductive phenology. Check List 9:973–979. 10.15560/9.5.973

[CR27] Hamer KC, Hill JK, Mustaffa N, Benedick S, Sherratt TN, Chey VK, Maryati M (2005) Temporal variation in abundance and diversity of butterflies in Bornean rain forest: opposite impacts of logging recorded in different seasons. J Trop Ecol 21:417–425. 10.1017/S0266467405002361

[CR28] Hartig F (2016) DHARMa: residual diagnostics for hierarchical (multi-level/mixed) regression models. R package version 0.1.0. https://CRAN.R-project.org/package=DHARMa

[CR30] Kerpel SM, Zacca T, Nobre CEB, Ferreira-Júnior A, Araújo MX, Fonseca A (2014) Capítulo 19: Borboletas do Semiárido: conhecimento atual e contribuições do PPBio. In: Bravo F, Calor A (eds) Artrópodes do Semiárido: biodiversidade e conservação. Printmídia, p 298

[CR31] Leal CRO, Silva JO, Sousa-Souto L, Neves FS (2016) Vegetation structure determines insect herbivore diversity in seasonally dry tropical forests. J Insect Conserv 20:979–988. 10.1007/s10841-016-9930-6

[CR32] Lima JNR, Zacca T (2014) Lista de espécies de borboletas (Lepidoptera: Hesperioidea e Papilionoidea) de uma área de semiárido na região Nordeste do Brasil. EntomoBrasilis 7:33–40. 10.12741/ebrasilis.v7i1.351

[CR33] Lourenço GM, Soares GR, Santos TP, Dáttilo W, Freitas AVL, Ribeiro SP (2019) Equal but different: natural ecotones are dissimilar to anthropic edges. PLoS One 14:e0213008. 10.1371/journal.pone.0213008 30830927 10.1371/journal.pone.0213008 PMC6398848

[CR35] MacArthur RH, Wilson EO (1967) The theory of island biogeography. Princeton University Press, Princeton

[CR36] Martinez-Adriano CA, Diaz-Castelazo C, Aguirre-Jaimes A (2018) Flower-mediated plant-butterfly interactions in an heterogeneous tropical coastal ecosystem. PeerJ 6:e5493. 10.7717/peerj.5493 30210938 10.7717/peerj.5493 PMC6130237

[CR37] Mittelbach G (2012) Community ecology. Sinauer Associates, MA

[CR38] Morais HC, Diniz IR, Silva DMS (1999) Caterpillar seasonality in a central Brazilian cerrado. Rev Biol Trop 47:1025–1033

[CR39] Morellato LPC, Alberti L, Hudson IL (2010) Applications of circular statistics in plant phenology: a case studies approach. In: Hudson I, Keatley M (eds) Phenological research. Springer, Dordrecht. 10.1007/978-90-481-3335-2_16

[CR40] Neves FS, Silva JO, Espírito-Santo MM, Fernandes GW (2014) Insect herbivores and leaf damage along successional and vertical gradients in a tropical dry forest. Biotropica 46:14–24. 10.1111/btp.12068

[CR42] Nobre CEB, Iannuzzi L, Schlindwein C (2012) Seasonality of fruit-feeding butterflies (Lepidoptera, Nymphalidae) in a Brazilian semiarid área. Int Sch Res Netw Zool 1–8. 10.5402/2012/268159

[CR44] Novais SMA, Monteiro GF, Macedo-Reis LE, Leal CRO, Neves FS (2019) Changes in the insect herbivore fauna after the first rains in a tropical dry forest. Oecol Aust 23:381–387. 10.4257/oeco.2019.2302.16

[CR45] Oksanen J, Blanchet FG, Kindt R, Legendre P, Minchin PR, O’Hara RB et al (2013) Package vegan: community ecology package. R package version 2.0–10. https://cran.r-project.org/web/packages/vegan/vegan.pdf

[CR46] Oliveira LM, Sousa RM, Correa NER, Santos AF, Giongo M (2018) Florística e síndromes de dispersão de um fragmento de Cerrado ao sul do estado do Tocantins. Scientia Agrar Paranaensis 104–111

[CR47] Orlandin E, Piovesan M, Carneiro E (2020) Borboletas do Meio-Oeste de Santa Catarina: história natural e guia de identificação. Edição Independente, Joaçaba

[CR48] Palo-Jr H (2017) Borboletas do Brasil. Editora Vento Verde, São Paulo

[CR49] Pennington RT, Lehmann CER, Rowland LM (2018) Tropical savannas and dry forests. Curr Biol 28:527–548. 10.1016/j.cub.2018.03.014 10.1016/j.cub.2018.03.014 29738723

[CR51] Pessoa MS, Hambuckers A, Benchimol M, Rocha-Santos L, Bomfim JA, Faria D, Cazetta E (2017) Deforestation drives functional diversity and fruit quality changes in a tropical tree assemblage. Perspect Plant Ecol Evol Syst 28:78–86. 10.1016/j.ppees.2017.09.001

[CR65] Pezzini FF, Ranieri, BD, Brandão D, Fernandes GW, Quesada M, Espírito-Santo MM et al (2014) Changes in tree phenology along natural regeneration in a seasonally dry tropical forest. Plant Biosyst 148: 965–974. 10.1080/11263504.2013.877530

[CR71] R Core Team (2020) R: a language and environment for statistical computing. R foundation for statistical computing, Vienna. Available at: http://www.R-project.org

[CR53] Ribeiro DB, Batista R, Prado PI, Brown JK, Freitas AV (2012) The importance of small scales to the fruit-feeding butterfly assemblages in a fragmented landscape. Biodivers Conserv 21:811–827. 10.1007/s10531-011-0222-x

[CR54] Rodrigues PMS, Schaefer CE, Silva JO, Ferreira-Junior WG, Santos RM, Neri AV (2018) The influence of soil on vegetation structure and plant diversity in different tropical savannic and forest habitats. J Plant Ecol 11:226–236. 10.1093/jpe/rtw135

[CR56] Santos JP, Iserhard CA, Carreira JYO, Freitas AVL (2017) Monitoring fruit-feeding butterfly assemblages in two vertical strata in seasonal Atlantic Forest: temporal species turnover is lower in the canopy. J Trop Ecol 33:345–355. 10.1017/S0266467417000323

[CR57] Santos LN, Kerpel SM, Medeiros AD, Brito MRM (2023) Borboletas do Nordeste: as borboletas em áreas protegidas de florestas nordestinas. EDUFCG, Campina Grande

[CR60] Silva JMC, Leal IR, Tabarelli M (2017b) Caatinga: the largest tropical dry forest region in South America. Springer International Publishing, Cham

[CR58] Silva JO, Espírito-Santo MM, Melo GA (2012) Herbivory on *Handroanthus ochraceus* (Bignoniaceae) along a successional gradient in a tropical dry forest. Arthropod-Plant Interact 6:45–57. 10.1007/s11829-011-9160-5

[CR61] Silva JO, Espírito-Santo MM, Santos J, Rodrigues PMS (2020) Does leaf flushing in the dry season affect leaf traits and herbivory in a tropical dry forest? Sci Nat 107:51. 10.1007/s00114-020-01711-z 10.1007/s00114-020-01711-z 33241430

[CR59] Silva JO, Leal CRO, Espírito-Santo MM et al (2017a) Seasonal and diel variations in the activity of canopy insect herbivores differ between deciduous and evergreen plant species in a tropical dry forest. J Insect Conserv 21:667–676. 10.1007/s10841-017-0009-9

[CR62] Spaniol RL, Duarte LS, Mendonça-Jr MS, Iserhard CA (2019) Combining functional traits and phylogeny to disentangling Amazonian butterfly assemblages on anthropogenic gradients. Ecosphere 10:28–37. 10.1002/ecs2.2837

[CR66] Wolda H (1988) Insect seasonality, why? Ann Rev Ecol Syst 19:1–18

[CR63] Zacca T, Bravo F (2012) Borboletas (Lepidoptera: Papilionoidea e Hesperioidea) da porção norte da chapada Diamantina. Bahia Brasil Biota Neotrop 12:117–126. 10.1590/S1676-06032012000200012

