# Supplementary material for: Vertical stratification and seasonality of fruit-feeding butterfly diversity in a Neotropical dry forest
Source: Naturwissenschaften. 2026 May 13;113(3):61. doi: 10.1007/s00114-026-02107-1 (PMC13171922; doi:10.1007/s00114-026-02107-1)
Supplement: Supplementary file 1 — Supplementary Material 1 (DOCX 1.54 MB) [file 114_2026_2107_MOESM1_ESM.docx]

**Supplementary Material**

**The Role of Vertical Stratification and Climate on Fruit-Feeding Butterfly Diversity in a Seasonally Dry Forest**

The Science of Nature

Bianca Santana Dias Nascimento^1^, Gabriela de Araújo Silva^1^, Uriel de Jesus Araújo Pinto^2^, João Rafael Macêdo^2^, Marina do Vale Beirão^3^, Jhonathan de Oliveira Silva^1,2^

^1^ Universidade Estadual de Feira de Santana, Programa de Pós-Graduação em Ecologia e Evolução, Feira de Santana, Bahia, Brazil

^2^ Universidade Federal do Vale do São Francisco, Colegiado de Ecologia. Senhor do Bonfim, Bahia, Brazil

^3^ Laboratório de Ecologia de Insetos, Universidade Federal de Minas Gerais, Belo Horizonte, MG, Brazil

Correspondence to: jhonathanos@gmail.com

| **Fournier Scale** | |
| --- | --- |
| **Categories** | **Description** |
| 0 | Absence of phenophase |
| 1 | Presence of phenophase with magnitude from 1% to 25% |
| 2 | Presence of phenophase with magnitude from 26% to 50% |
| 3 | Presence of phenophase with magnitude from 51% to 75% |
| 4 | Presence of phenophase with magnitude from 76% to 100% |

**Table S1.** Description table of the Fournier Intensity Percentage.


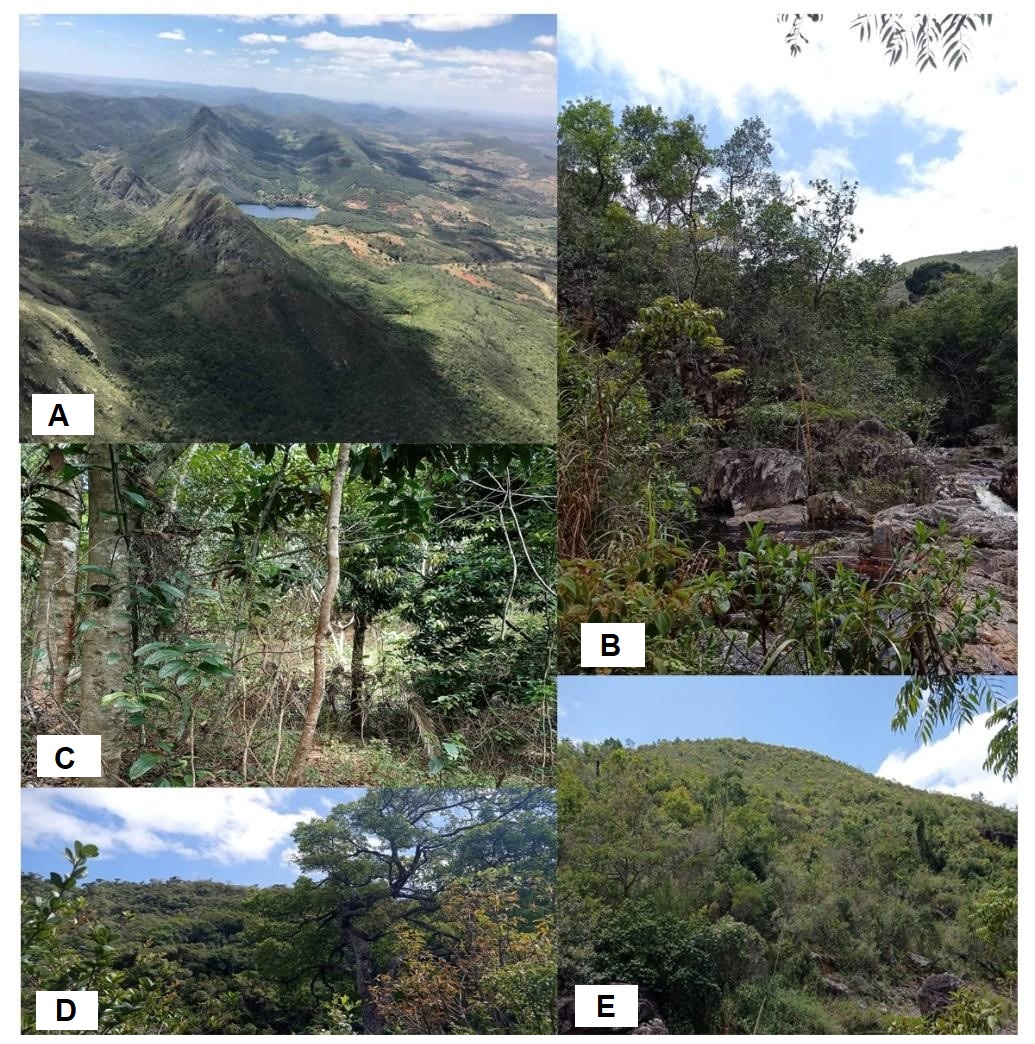


**Fig. S1** Seasonally dry tropical forest (TDF) located in Serra da Bananeira, belonging to the Serra de Jacobina complex, northern portion of the Espinhaço chain, Bahia. A- Aerial view of Serra da Bananeira; B – riparian vegetation and Aipim River; C, D, and E – TDF vegetation during the rainy season


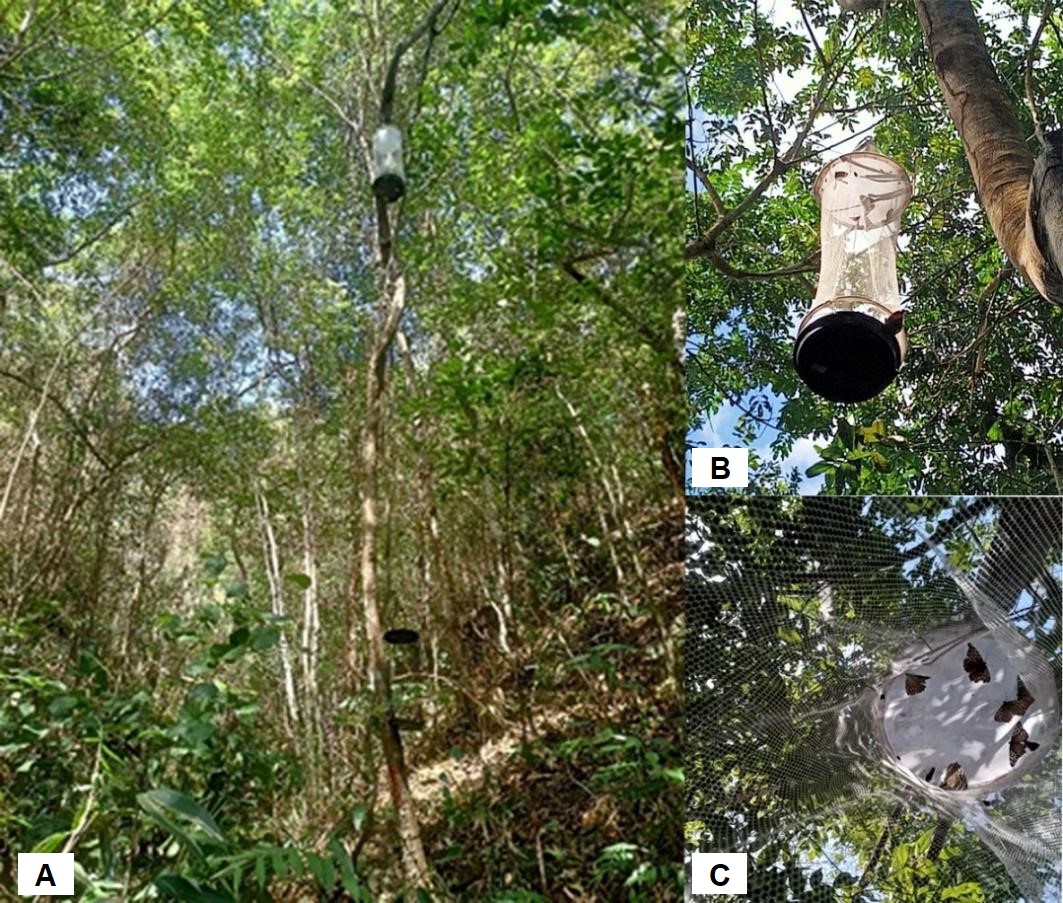


**Fig. S2** A- Van Someren-Rydon traps installed in the understory and canopy to collect fruit-feeding butterflies; B – butterfly entering the canopy trap; C- trap in the understory with captured butterflies


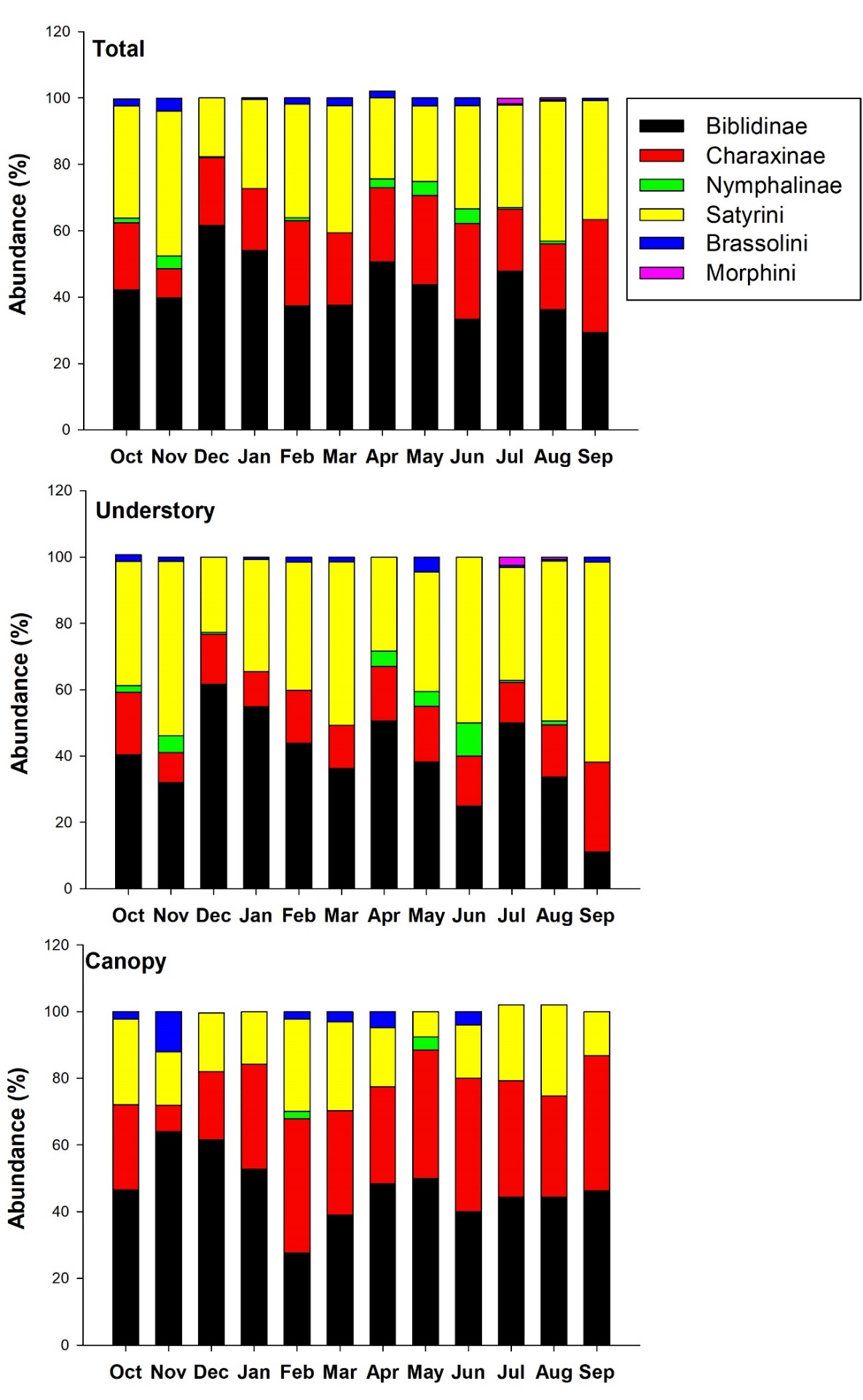


**Fig. S3** Proportion of abundance of Nymphalidae subfamilies/tribes in the community and by habitats (understory and canopy) over a year, in Serra da Bananeira, Bahia, Brazil

**
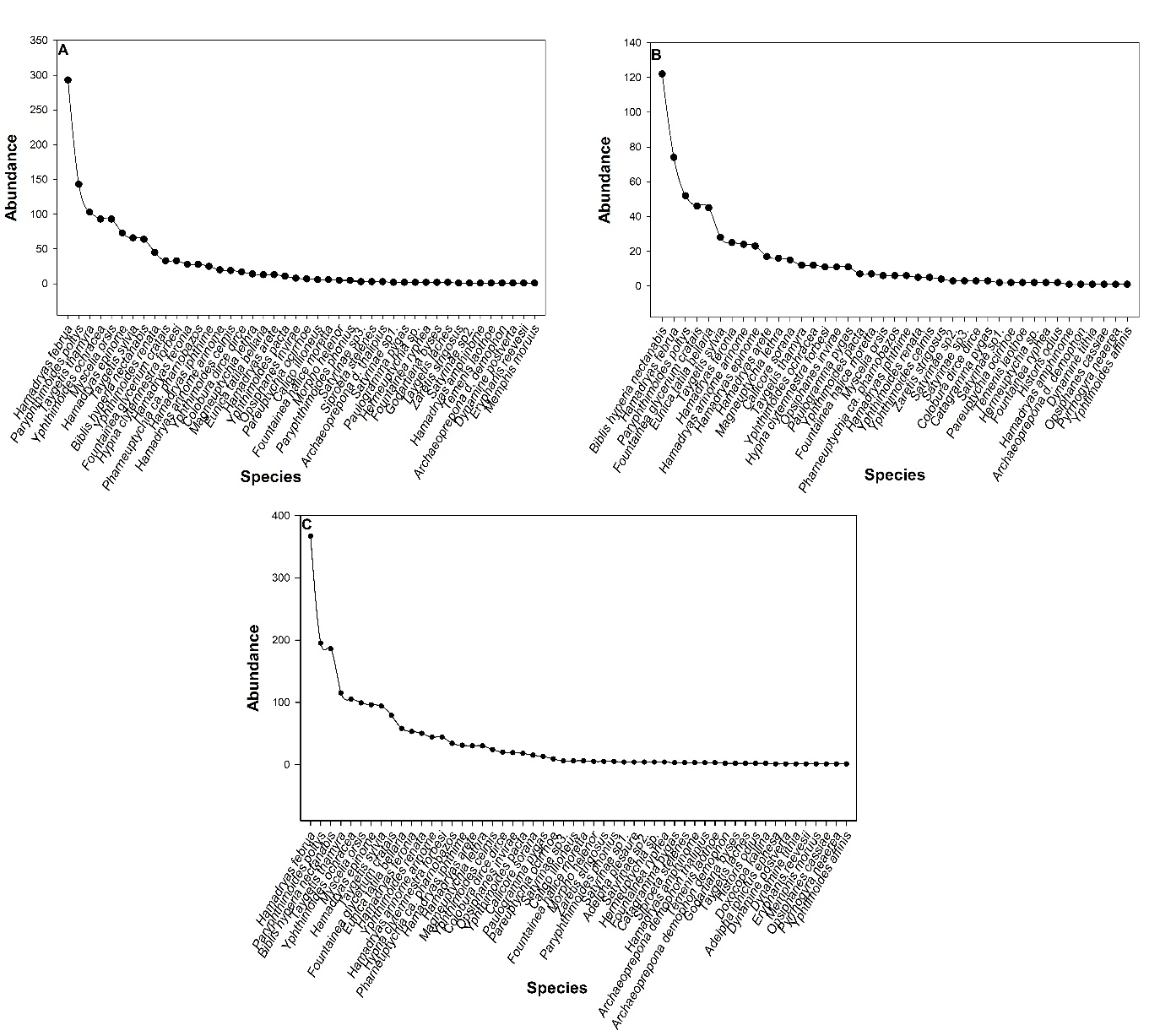
**

**Fig. S4** Abundance rank demonstrating the variation in species composition of the (A) understory and (B) canopy and (C) total community
